# Characterization of Antimicrobial Resistance Dissemination across Plasmid Communities Classified by Network Analysis

**DOI:** 10.3390/pathogens3020356

**Published:** 2014-04-15

**Authors:** Akifumi Yamashita, Tsuyoshi Sekizuka, Makoto Kuroda

**Affiliations:** Pathogen Genomics Center, National institute of infectious diseases, 1-23-1 Toyama, Shinjuku-ku, Tokyo 162-8640, Japan; E-Mails: uhmin@niid.go.jp (A.Y.); sekizuka@niid.go.jp (T.S.)

**Keywords:** plasmidome, network analysis, horizontal gene transfer, Inc replicon, antimicrobial resistance, ESBL, carbapenemase

## Abstract

The global clustering of gene families through network analysis has been demonstrated in whole genome, plasmid, and microbiome analyses. In this study, we carried out a plasmidome network analysis of all available complete bacterial plasmids to determine plasmid associations. A blastp clustering search at 100% aa identity cut-off and sharing at least one gene between plasmids, followed by a multilevel community network analysis revealed that a surprisingly large number of the plasmids were connected by one largest connected component (LCC), with dozens of community sub-groupings. The LCC consisted mainly of *Bacilli* and *Gammaproteobacteria* plasmids. Intriguingly, horizontal gene transfer (HGT) was noted between different phyla (*i.e.*, *Staphylococcus* and *Pasteurellaceae*), suggesting that *Pasteurellaceae* can acquire antimicrobial resistance (AMR) genes from closely contacting *Staphylococcus* spp., which produce the external supplement of V-factor (NAD). Such community network analysis facilitate displaying possible recent HGTs like a class 1 integron, *str* and *tet* resistance markers between communities. Furthermore, the distribution of the Inc replicon type and AMR genes, such as the extended-spectrum ß-lactamase (ESBL) CTX-M or the carbapenemases KPC NDM-1, implies that such genes generally circulate within limited communities belonging to typical bacterial genera. Thus, plasmidome network analysis provides a remarkable discriminatory power for plasmid-related HGT and evolution.

## 1. Introduction

Bacterial plasmids are self-replicating, extrachromosomal replicons that are key agents of change in microbial populations [1]. It is well known that plasmid vectors are frequently transmitted from one bacterium to another [2,3,4,5]. Plasmids, as well as conjugative transposons, are crucial mediators of horizontal gene transfer (HGT), a process that significantly influences bacterial activity and evolution. Most antimicrobial resistance (AMR) genes can be acquired through plasmid-mediated HGT [2]. The increased number of AMR genes acquired by pathogenic bacteria threatens to return us to the pre-antibiotics era. The misuse of antimicrobial medications accelerates this natural phenomenon. Moreover, poor infection control practices have encouraged the spread of AMR. Thus far, it has been challenging to reveal the global dissemination of AMR plasmids by molecular genotyping. Molecular genotyping techniques, such as MLST, MLVA, and IS-typing, are available to hospitals, communities, and global organisations for tracing clonal bacteria dissemination. Furthermore, next-generation sequencing (NGS) can identify bacterial strain-specific genetic markers including single nucleotide polymorphisms, facilitating the identification of disseminated pathogenic bacteria clones, such as *Staphylococcus aureus* [6], *Clostridium difficile* [7], *Acinetobacter baumannii* [8], and *Mycobacterium tuberculosis* [9].

AMR can be widely transferred among different strains and species from closely related taxonomic classes [2,3,10]. Indeed, the extended-spectrum ß-lactamase (ESBL) CTX-M and several carbapenemases have been widely identified in many species of Enterobacteriaceae [11], indicating that a molecular characterisation of plasmid sequences would be more beneficial for tracing the transmission of AMR than would characterising whole genome sequences, because most AMR genes can be acquired through plasmid-mediated HGT [2]. As whole genome sequencing of a strain harbouring multiple plasmids is not able to characterize single source of plasmid genetic feature, sequencing only plasmid is rather simple and cost effective.

Previous research has investigated global clustering of gene families using network analysis, as well as global network analysis of DNA families shared among cellular, plasmid, and phage genomes [12]. Similar studies focusing on plasmids showed frequent HGT over geographical habitat or taxonomical barrier [13,14]. The evolutionary dynamics of plasmids in *Acinetobacter* spp. (pan-plasmidome analysis) have also been examined [14]. Furthermore, both microbiome and plasmidome analyses have been performed for the bovine rumen [15]. Plasmidome analysis in particular has been carried out for ESBL in *Escherichia coli* [16] and various plasmids in *Enterococcus faecalis* [17]. Module-based phage network analysis has been demonstrated to suggest a reticulate representation of evolutionary and functional relationships between phage genomes [18].

These intensive studies focused on comprehensive network analysis using relatively lower gene similarity (~95%), thus, we performed a network analysis at 100% aa identity cut-off and sharing at least one gene between plasmids, leading to characterize the most recently occurred HGT or plasmid transfer. Such strict parameters will facilitate a better understanding of the current and future prospects of AMR dissemination based on the plasmid transfer.

## 2. Results and Discussion

In total, 3793 complete plasmid sequences were selected from the NCBI database. Accordingly, 275,954 protein sequences were extracted (downloaded on 10 June, 2013).

### 2.1. Number of Edges and Nodes between Each Plasmid

**Figure 1:**
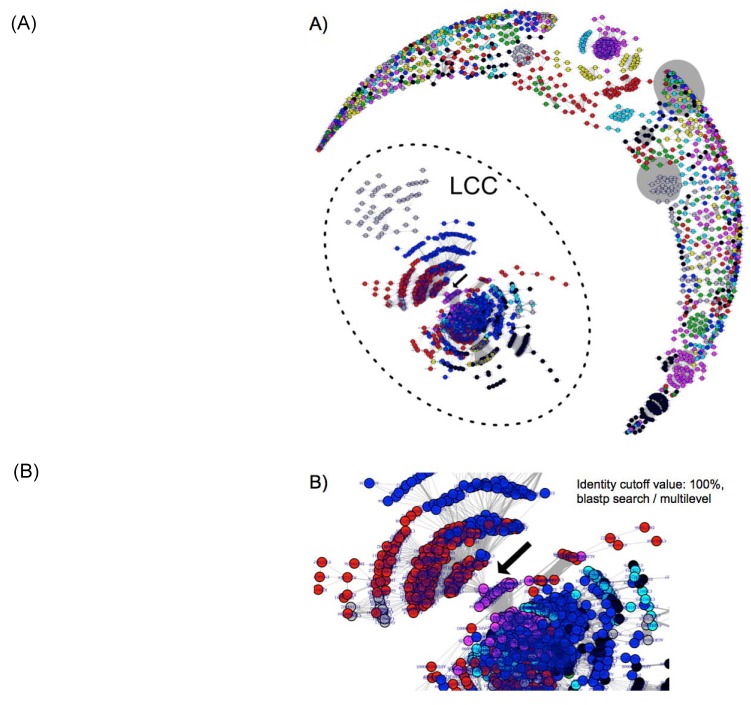
Plasmidome network analysis. (**A**) The filled circles (nodes) represent the respective plasmids. The grey lines (edges) represent the connections among the plasmids. The thickness of the edges represents the number of genes shared among the plasmids. The edges denoting large numbers of shared genes appear as grey ellipses. The colours of the nodes are automatically set according to the plasmid communities inferred from the multilevel method using the R igraph package. The nodes within the dotted circle represent the largest connected component (LCC). (**B**) Close-up of the connecting points between *Gammaproteobacteria* and *Bacilli*.

Plasmids that share at least one homologous sequence were extracted as connected nodes based on either a blastp or uclust homology search of coding sequences in each plasmid. As shown in Table 1, the number of connected-nodes decreases as the identity cut-off value increases, indicating that lower thresholds generate more connected nodes. The single connected components (CCs) are composed of all of the plasmids (nodes) with at least a single connection to others. The number of CCs is always slightly larger for uclust than it is for blastp, suggesting that blastp is a more rigorous clustering search method for finding the homologous lineages of ORFs following CC assignment. Thus, we performed blastp searches for the subsequent plasmidome network analysis. We obtained an image of the plasmidome network using a 100% identity cut-off value (Figure 1A).

**Table 1:** Number of connected component and community according to identity cutoff values.

Clustering program	Identity cutoff (%)	Edges	Connected nodes	Connected components	Nodes in LCC	LCC % in total	Number of communities in the LCC using multilevel method
BLASTP	50	251,961	3529	57	3265	86.1%	19
BLASTP	60	177,932	3444	70	2793	73.6%	25
BLASTP	70	129,545	3358	90	2613	68.9%	31
BLASTP	80	104,815	3229	127	2265	59.7%	26
BLASTP	90	81,752	3060	162	1995	52.6%	34
BLASTP	99	52,029	2633	236	1389	36.6%	36
BLASTP	100	41,956	2496	259	1241	32.7%	26
UCLUST	50	188,844	3524	71	3233	85.2%	ND
UCLUST	60	147,899	3435	75	2762	72.8%	ND
UCLUST	70	121,841	3349	98	2558	67.4%	ND
UCLUST	80	101,902	3213	133	2241	59.1%	ND
UCLUST	90	82,046	3057	174	1949	51.4%	ND
UCLUST	99	52,578	2626	240	1369	36.1%	ND
UCLUST	100	43,275	2501	263	1232	32.5%	ND

CC: connected component; LCC: largest connected component; ND: not determined.

### 2.2. Connected Components (CCs)

The largest CC (LCC) contains 32.7% of all the investigated plasmids, even after increasing the identity cut-off value to 100% (Figure 1A, Table 1). Although this network may contain identical plasmid pairs by means of vertical inheritance or plasmid transfer, the existence of such a large LCC from vast variety of hosts (will be discussed in the next section) clearly indicates that most of the plasmids share genes by HGT or plasmid transfer, suggesting that a frequent and ongoing dissemination of genes occurs between plasmids. To detect most recently occurred HGTs (not just phylogenetic relationships) we selected a threshold with identity cut-off value of 100% for further analysis. Under this condition 1297 plasmids from many kinds of bacterial species remain unconnected. As well as the previous study, the LCC showed power law distribution of the degree measures of nodes, which comes from the “scale-free” network feature [19,20]. Accordingly, the LCC mostly is composed of Enterobacteriaceae, such as Escherichia, Klebsiella, and Salmonella, corresponding to the result in Tamminen *et al.* [13].

### 2.3. Community Network Analysis

The obtained LCC contains large number of nodes for further representing the characteristic network structure, thus, we subdivided the LCC into several “communities”, using available clustering methods in the R igraph package [21] including the fastgreedy, multilevel, edge betweenness, walktrap, label propagation, and infomap methods. To estimate the best method for community analysis, we employed the Shannon index [22], which estimates the diversity index of the host bacterial lineage. We selected the method that provided the set of communities with the lowest Shannon index.

**Figure 2:**
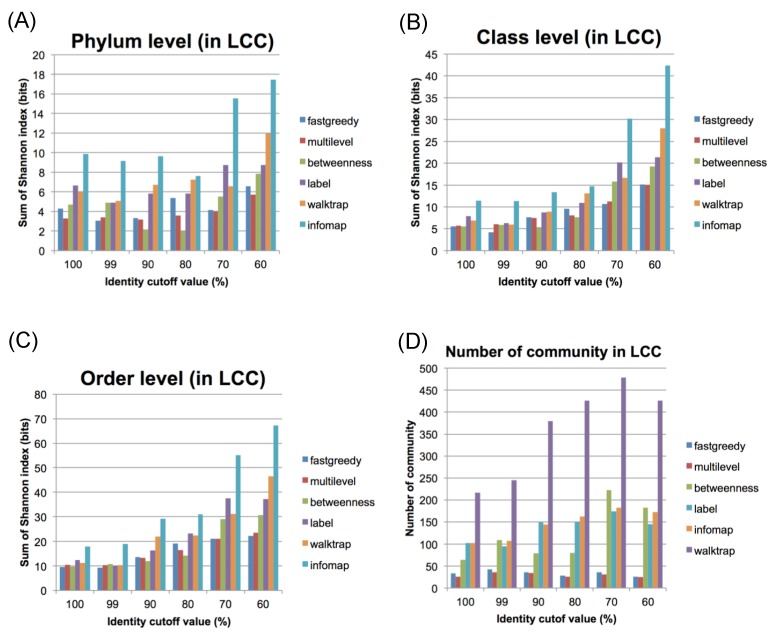
Evaluation of community connections using several methods. The Shannon index values for the LCC using different identity cut-off values and community estimation methods at the phylum (**A**), class (**B**), and order (**C**) levels. (**D**) The numbers of communities in the LCC using different identity cut-off values.

**Figure 3:**
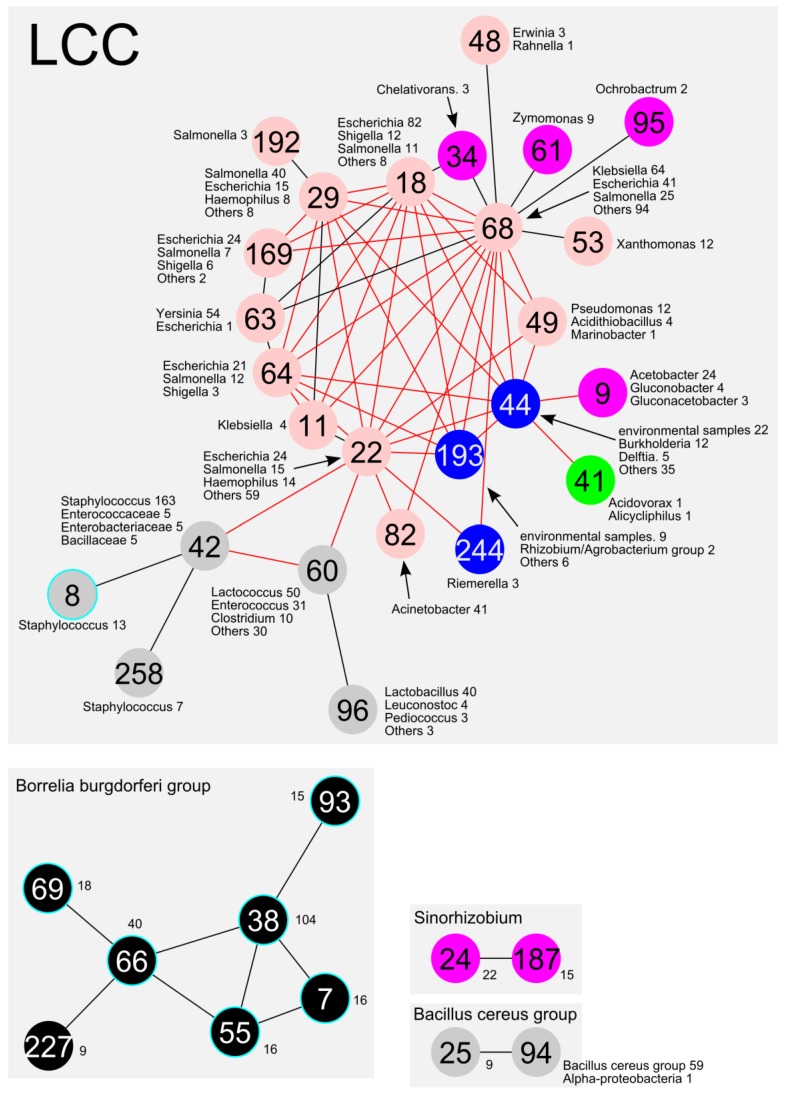
A schematic representation of the communities from the whole image shown in Figure 1. The communities mainly included *Alphaproteobacteria*, *Betaproteobacteria*, *Gammaproteobacteria*, *Bacilli*, and *Borrelia*, which are indicated by purple, green, pink, grey, and black circles, respectively. The other communities are indicated by blue circles. The number of the top three species in the community is shown beside the circle. The circle with the light-blue rim represents the communities without AMR genes. The red lines represent the AMR gene related connections among the communities.

As shown in Figure 2, the fastgreedy, multilevel and betweenness methods generated relatively better results, with lower diversity index values at every identity cut-off level for distinct taxonomic levels (Figure 2A: phylum, 2B: class, 2C: order). Accordingly, the fastgreedy and multilevel methods generated significantly fewer communities in the LCC (Figure 2D). The multilevel method, however, was considered a better choice at every identity cut-off. Thus, we decided to employ the multilevel method in the subsequent analysis because it obtained the fewest communities. Based on the multilevel method, a total of 291 communities (e.g., co68 …) were extracted from all CCs at an identity cut-off of 100%, while the LCC can be well subdivided into 26 communities (Table 1 and Figure 3).

**Figure 4:**
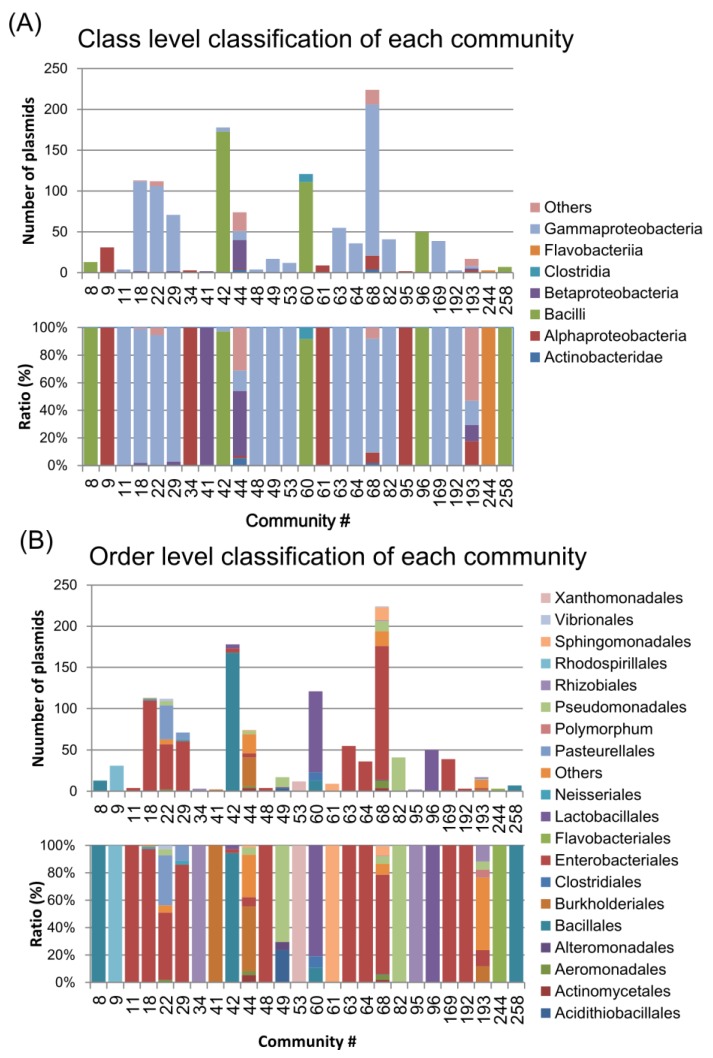
The host bacterial components of the communities. The upper and lower graphs show the numbers and ratios of the host bacterial components, respectively, for the class (**A**) and order (**B**) levels.

Most of the communities in the LCC consisted of plasmids from the *Gammaproteobacteria, Alphaproteobacteria, Betaproteobacteria* and *Bacilli* classes (Figure 4A) or *Enterobacteriales and Bacillales* orders (Figure 4B). For instance, co68 was mostly composed of *Enterobacteriales,* such as *Klebsiella*, *Escherichia*, and *Salmonella* spp. (Figure 3 and Figure 4B). In addition, co22 and co29 were composed of *Enterobacteriales* such as *Escherichia*, *Salmonella* and *Haemophilus* spp. (Figure 3 and Figure 4B). Some specific bacterial plasmids were predominant in smaller communities (e.g., *Yersinia* in co63, *Acinetobacter* in co82, *Staphylococcus* in co8 and co258, and *Xanthomonas* in co53).

Clustering coefficient values in any communities in LCC was estimated as median 0.681 (SD: 0.125), indicating that no community consisted of homogenous plasmids. The community network analysis suggests that bacterial plasmids can be grouped into communities according to biologically relevant features.

### 2.4. Connections between Communities

#### 2.4.1. Genes Involved in the Connections between Communities

**Figure 5:**
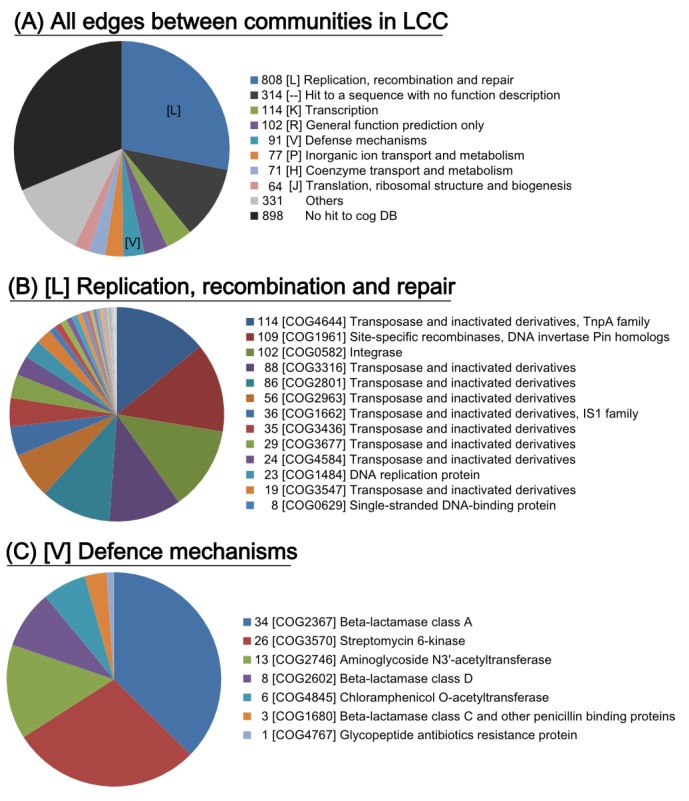
COG annotation for genes on the edges between communities. (**A**) COG categories on all of the edges in the LCC. (**B**) The COG ID for the category [L] of replication, recombination and repair. (**C**) The COG ID for the category [V] of defence mechanisms.

The abovementioned multilevel community analysis suggests a crucial linkage among the 26 communities in LCC. Thus, we searched for genes involved in the unique connections between communities. Blast search against the COG database revealed that the “[L] Replication, recombination and repair” subgroup was the predominantly detected at 60.0% (2283 of 3806) as transposases on the edges (Figure 5), indicating that multiple IS-related transposases could be involved in HGT between communities. We found that most of the edges were related to antimicrobial resistance genes (see the red line edges between the communities in Figure 3), suggesting that the “[V] defence mechanism” category could play a crucial role in the connection for HGT due to antimicrobial selective pressure (Figure 5). This result suggests that genetic exchange events between communities could be driven by genes related to replication/recombination/repair functions (COG [L]), as well as genes related to AMR.

#### 2.4.2. Frequently Identified Gene Combination among the Edges between Communities

**Figure 6:**
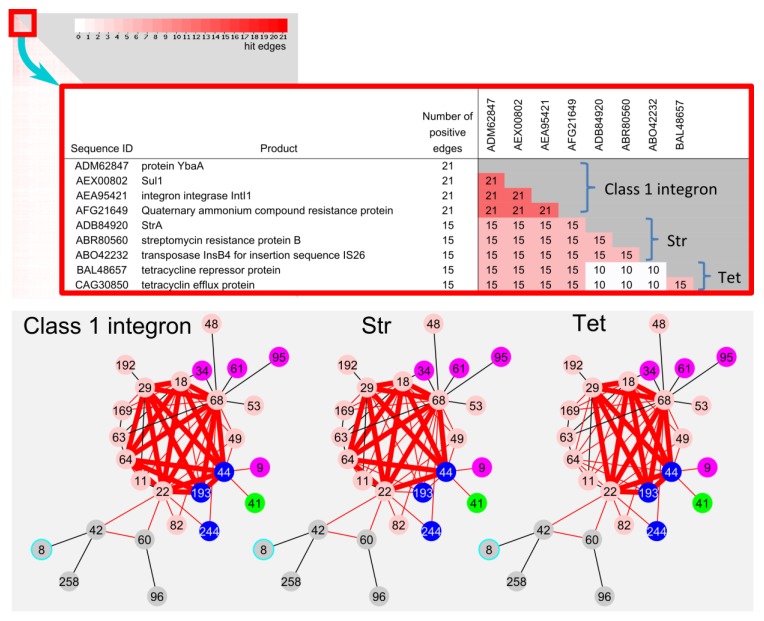
Frequently hit sequence on the 66 edges in LCC. Heatmap of sequence pairs of 1506 sequences on the 66 edges in LCC. Close-up of top 9 frequently hit sequences. Class 1 integron (21 edges), *str* (15 edges) and *tet* (15 edges) genes on the edges were shown in bold red line.

Since shared genes on the edge between communities could be a possible candidate for recent HGT genes, frequently shared gene was extracted from the total non-redundant 1506 genes on the 66 edges in LCC. The combination of ybaA, sul1, intI1 and qacEΔ1 were most frequently identified on 21 edges (Figure 6). This combination has been well characterized as class 1 integron, which is known as a major player of indiscriminate dissemination of AMR [23]. In addition, the combinations of “insB, strA, and strB” (streptomycin resistance marker) and “tetR and tetA” (tetracycline resistance marker) were also frequently found on 15 edges, respectively. The str and tet combinations were not found on the co193 and co 64-related edges, respectively.

In addition, we confirmed whether class 1 integron could be transmitted *en bloc.* The basic components of class 1 integrons consist of IntI1, QacEdelta1, Sul1 and YbaA (Table 2: 01-02/03-04-05). Forty plasmids from the 3793 complete plasmid sequences in this study have been identified to carry the contiguous components of a class 1 integron. Further investigation suggested that additional AMR genes are integrated into the class 1 integron as cassette gene (Table 2). A class 1 integron group including additional aminoglycoside resistance (01-12-02-04-05) was found in communities co18 (plasmid: DQ364638 and AP000342), co29 (plasmid: AB605179), and co68 (plasmid: HQ201416). Furthermore, class 1 integrons with integrated trimethoprim-resistant dihydrofolate reductase (01-36-12-02-04-05) was found in communities co29 (FN432031) and co64 (JX566770). These results strongly suggest that complete class 1 integrons together with additional integrated AMR genes could be transmitted among communities*.* Such AMR markers could play a crucial role for recent HGT, leading to contribute to the vast linkages among variable plasmid communities as the global dissemination of AMR.

**Table 2:** The class 1 integron mediated en bloc transmission of AMR genes among communities.

Order of gene groups	Community								Total
	co18	co22	co29	co44	co64	co68	co193		
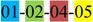						1			1
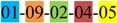		1							1
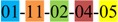		1							1
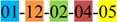	2		1			1			4
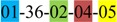						1			1
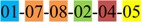							1		1
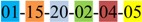		1							1
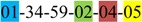						2			2
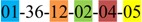			1		1				2
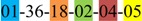						1			1
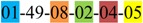				2					2
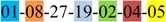						1			1
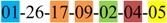						1			1
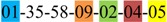						1			1
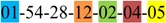						1			1
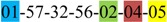						1			1
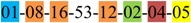	1								1
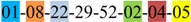						1			1
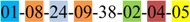						1			1
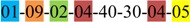						1			1
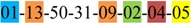		1							1
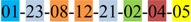						1			1
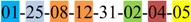						1			1
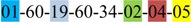						1			1
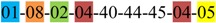						1			1
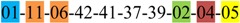		1							1
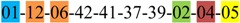		3							3
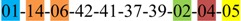		1							1
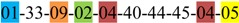						1			1
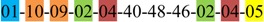						1			1
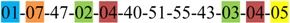						1			1
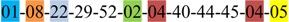						1			1
Total	3	9	2	2	1	22	1		40
**Gene group**	**Products**								
01	integron integrase IntI1.							class 1 integron component	
02, 03	quaternary ammonium compound-resistance protein QacEdelta1.								
04	sulphonamide resistant dihydropteroate synthase Sul1.								
05	protein YbaA.								
06	aminoglycoside 3'- *N*-acetyltransferase protein *aacC*.							aminoglycoside resistance	
07	aminoglycoside 3'- *N*-acetyltransferase.								
08	aminoglycoside acetyltransferase.								
09	aminoglycoside adenylyltransferase.								
10	aminoglycoside *N*(6')-acetyltransferase.								
11–14	aminoglycoside resistance protein *aadA*.								
15	aminoglycoside-2'-adenylyltransferase.								
16	acetyltransferases *aac(3)-Ia*.								
17	putative aminoglycoside 6'- *N*-acetyltransferase.								
18	putative aminoglycoside adenyltransferase.								
19	class D β-lactamase OXA-10.							β-lactamase	
20	class D β-lactamase OXA-21.								
21	class D β-lactamase OXA-2.								
22	class D β-lactamase OXA.								
23	metallo-β-lactamase GIM-1.								
24	metallo-β-lactamase IMP-6.								
25	metallo-β-lactamase VIM-1.								
26	chloramphenicol acetyltransferase catB2.							chloramphenicol resistance	
27	chloramphenicol acetyltransferase catB8.								
28, 29	chloramphenicol acetyltransferase.								
30	chloramphenicol aminotransferase.								
31	chloramphenicol resistance protein CmlA1.								
32	dihydrofolate reductase DfrA5.							dihydrofolate reductase	
33-35	dihydrofolate reductase.								
36	trimethoprim-resistant dihydrofolate reductase type I DhfrA1.								
37–40	transposase.								
41	molecular chaperone GroEL.								
42	molecular chaperone GroES.								
43	fluoroquinolone resistance protein QnrB2.								
44	quinolone-resistance determinant Qnr.								
45–52	Others								
53-60	hypothetical protein.								

#### 2.4.3. Connections between Plasmid Communities from Different Divisions

The community network analysis revealed unique linkages between *Bacilli* (co42 and co60) and *Gammaproteobacteria* (co22) (Figure 1B and Figure 3). The plasmids from *Staphylococcus* and *Pasteurellaceae* were connected by two plasmids from *Pasteurella multocida* (pCCK411, GenBank_ID: FR798946) and *Haemophilus parasuis* (pQY431, GenBank_ID: KC405065) (Figure 1B).

**Figure 7:**
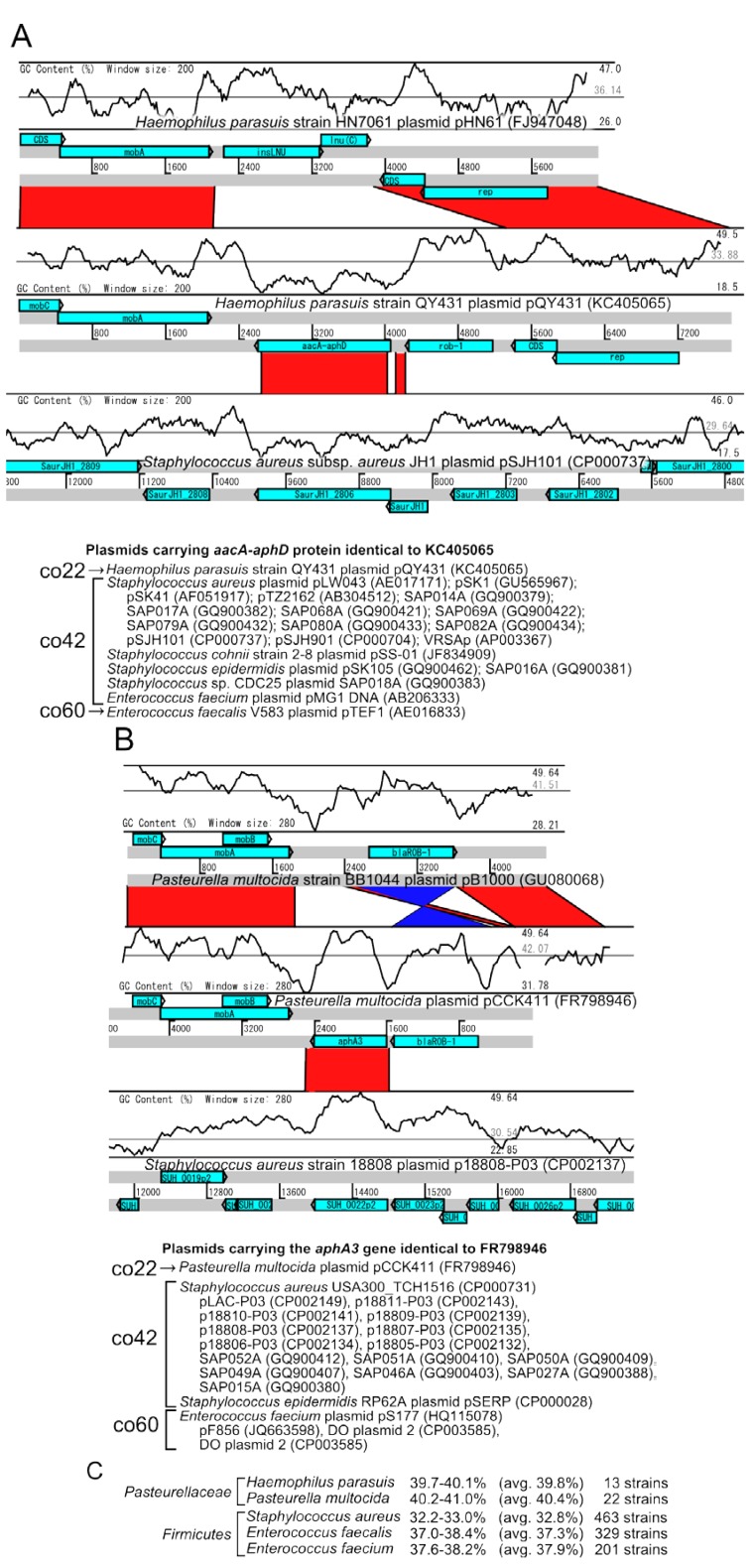
Pairwise comparisons of the (**A**) *Haemophilus parasuis* and (**B**) *Pasteurella multocida* plasmids carrying the *aacA-aphD* and *aphA3* genes compared to *Staphylococcus aureus* plasmids. The light-blue boxes represent the genes on the plasmids. The red and blue bars represent the homologous regions in the forward and inverted directions, respectively. The plasmids carrying the homologous genes are shown below the pairwise comparison. (**C **) Average GC-content of the host chromosomes.

The pQY431 in *H. parasuis* appeared to have acquired a bifunctional ACC/APH gene (*aacA-aphD*, AGK85216.1) from a plasmid in *Staphylococcus* spp., because GC-content of ACC/APH gene corresponds to the average of *S. aureus* plasmid, while it is lower than the average of *H. parasusis* plasmid. This plasmid also have a ß-lactamase (*bla*_ROB-1_, AGK85217) from another plasmid in *Pasteurellaceae* species (Figure 7A).

In addition, the pCCK411 plasmid in *P. multocida* shares a kanamycin resistance gene (*aphA3*, CBZ06037) with *Staphylococcus* plasmids and the *bla*_ROB-1_ gene from another *Pasteurellaceae* plasmids. Interestingly, higher GC content in this region in *Staphylococcus* suggests that this gene might be acquired from other plasmids such as *Pasteurella multocida* (Figure 7B). An additional blastp search with the NCBI nt database indicated that the identical sequences of the *aacA-aphD* and *aphA3* genes were detected in plasmids from the bacteria from the *Bacilli* class, such as *Staphylococcus*, or *Enterococcus* plasmids. 

Of particular note, *Haemophilus*/*Pasteurella* spp. required an external supplement of V-factor (NAD) from staphylococci for growth support [24]. In addition, the *Pasteurellaceae Hin* subclade (*H. influenzae, P. multocida*) possessed the natural competence and transformation ability [25]. These observations support the hypothesis that the *aacA-aphD* gene in *H. parasuis* (pQY431) was introduced through close contact with *Firmicutes* such as staphylococci. Contrary, there has been no evidence how *Staphylococcus* could acquire the *aphA3* gene, but the close relationship may have promoted gene exchange between plasmids originating from different phyla.

This study revealed high relationship between *Pasteurellales* and *Staphylococcus* (Figure 7), although previous report characterized the gene transfer between *Actinobacteridae* and *Gammaproteobacteria* [13]. Such difference generated from the difference of parameter threshold, *i.e.*, Tamminen *et al.* (2012) required at least five shared homologous sequencess and 95% aa identity to connect plasmids, while this study picked up plasmid connections from at least one shared homologous sequences at 100% aa identity, indicating more stringent threshold could display a potential recent HGT in this study.

### 2.5. Distribution of Inc Types among Plasmid Communities

**Figure 8:**
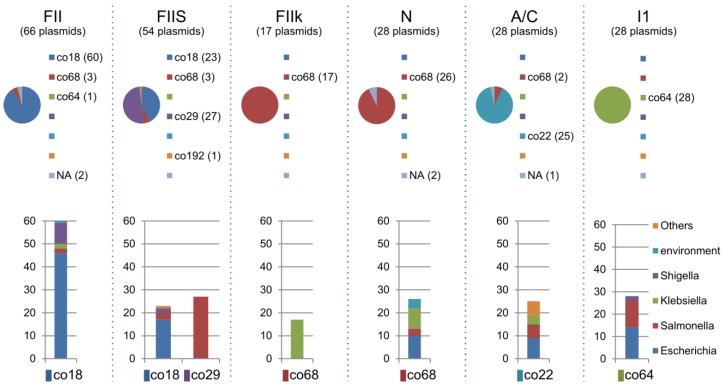
Components of the major Inc-type plasmids. The components of the communities of the major Inc-type plasmids and the bacterial components for each community are shown (Table 3).

Plasmids can be categorised by the plasmid replicon incompatibility group (Inc-group), using PCR-based methods to identify the replicons of the major plasmid families found in *Enterobacteriaceae* [26]. We performed *in silico* replicon typing for all of the plasmids against 22 known Inc groups (Table 3). The Inc types could be assigned to some plasmids in the LCC, and showed strong correlations between the community and the Inc type (e.g., IncFII: co18, FIIS: co18 and co29, FIIk: co68, N: co68, A/C: co22, I1: co64, and P: co193, in Table 3). IncFII-co18 and IncFIIS-co18 were found predominantly in *Escherichia*, while IncFIIk-co68 was found predominantly in *Klebsiella* and IncFIIS-co29 was found predominantly in *Salmonella* (Figure 8)*.* In contrast, IncA/C-co22 was composed of plasmids from various bacterial genera (Figure 8), suggesting that IncA/C has greater dissemination potential among *Enterobacteriacea*. IncN-co68 was closely related between *Escherichia* and *Klebsiella*. In addition, IncI1-co64 was closely related between *Escherichia* and *Salmonella*, suggesting that frequent potential transmissions can occur between these two bacterial genera. Interestingly, significantly larger amount of plasmids in both co22 and co68 could not be assigned for Inc type, implying the variety of Inc types remain to be elucidated.

**Table 3:** Distribution of Inc types and communities.

Inc type	Community							
	co11	co18	co22	co29	co64	co68	co192	co193
A/C	-	-	25	-	-	2	-	-
B/O	-	3	-	-	1	1	-	-
FIA	-	5	-	-	-	3	-	-
FIB	-	5	-	-	-	-	-	-
FIB-M	-	-	-	-	-	1	-	-
FII	-	60	-	-	1	3	-	-
FIIk	-	-	-	-	-	17	-	-
FIIS	-	23	-	27	-	3	1	-
HI1	-	1	-	-	-	1	-	-
HI2	-	-	-	-	-	5	-	-
HIB-M	3	-	-	-	-	-	-	-
I1	-	-	-	-	28	-	-	-
K	-	1	-	-	3	-	-	-
L/M	-	-	-	-	-	7	-	-
N	-	-	-	-	-	26	-	-
P	-	-	-	-	-	-	-	7
R	-	-	2	-	-	3	-	-
T	-	1	-	-	-	1	-	-
U	-	-	-	-	-	3	-	-
W	-	-	2	-	-	3	-	-
X1	-	1	-	7	-	-	-	-
X2	-	-	-	-	-	1	-	-
Not Assigned	1	13	83	37	3	144	2	10
Total	4	113	112	71	36	224	3	17

Shaded communities showed significant over-representation with *p*-value < 0.001 by chi-square test.

### 2.6. Distribution of AMR among Plasmid Communities

**Figure 9:**
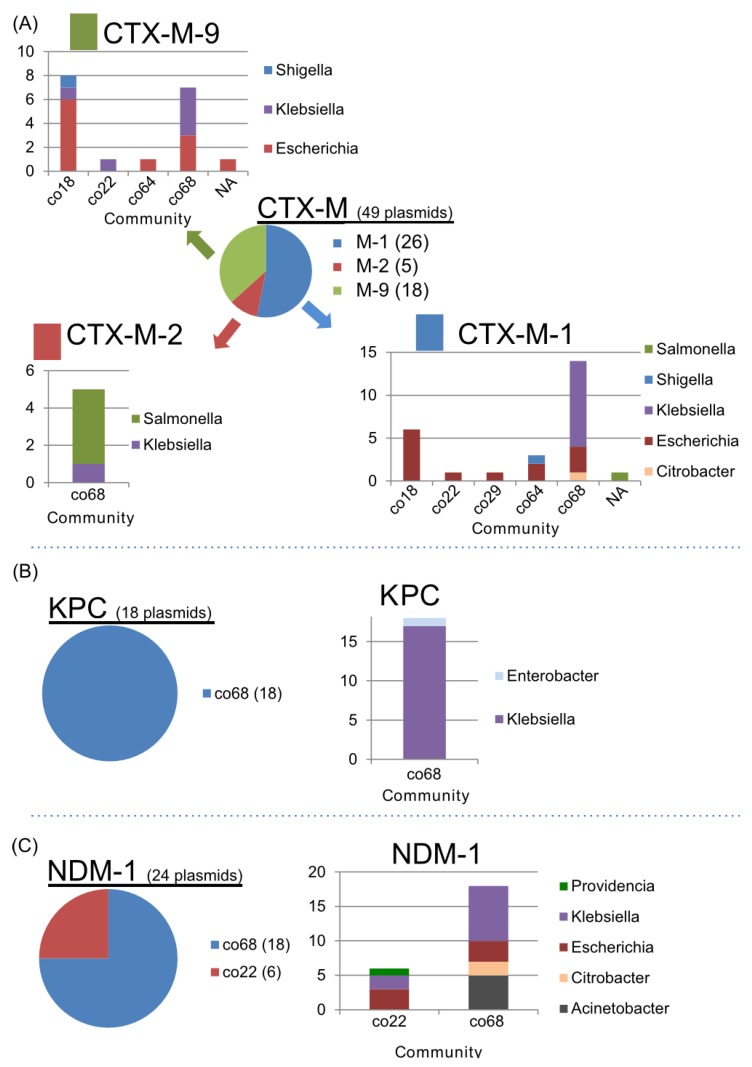
Components of the communities with AMR genes. (**A**) The CTX-M-1, -2 and -9 subgroups and the community and bacterial components. (**B**) KPC carbapenemase and the bacterial components. The plasmids carrying KPC are only found in co68. (**C**) The communities and bacterial components of the plasmids carrying the NDM-1 gene (Table 4).

Apart from the Inc-type related communities, the associations of AMR genes within communities were also investigated. CTX-M ß-lactamase is a broadly disseminated ESBL in *Enterobacteriacea* [27]. The CTX-M-1 and CTX-M-9 subgroups were broadly classified into multiple communities (co18, co22, co29, co64, and co68) (Figure 9A), while the CTX-M-2 subgroup was classified only as co68. More than 80% (40/49) of plasmids carrying the CTX-M ß-lactamase were found in co18 or co68. In addition, CTX-M-1 and CTX-M-9 were the most frequently found in the IncFII replicon plasmid (Table 4), suggesting that the dissemination of the CTX-M genes is linked to Inc replicon-dependent plasmid communities between co18 and co68 (Figure 3). Intriguingly, the abovementioned communities (co18, co22, co29, co64, and co68) were tightly connected to each other according to the community network analysis (Figure 3). Most of the plasmids carrying CTX-M-1 and CTX-M-9 were derived from *Escherichia* or *Klebsiella*, while most of the plasmids carrying CTX-M-2 were from *Salmonella* and *Klebsiella*.

As well as the class 1 integron, CTX-M ß-lactamases have been acquired through insertion sequence mediated transposition into a certain Enterobacterial plasmid with a possible natural competence from *Kluyvera* species [23,27]. The CTX-M-1 and -9 can be identified in multiple communities in this study (Figure 9A). Therefore, further dissemination of CTX-M-1 and -9 could be generated by plasmid transfer itself. In addition, the CTX-M-1 and -9 positive mobile element could transpose into other broad-host range plasmids, leading to wide dissemination of ESBL in Enterobacteriacea. On the other hand, CTX-M-2 subgroup is identified in only co68, suggesting that CTX-M-2 positive plasmid disseminates between *Salmonella* and *Klebsiella* species (Figure 9A).

The carbapenemases *K. pneumoniae* carbapenemase (KPC) and New Delhi metallo-ß-lactamase (NDM-1) were unique to the co22 and co68 communities (Figure 9B). KPC was only found in co68, mainly in IncFIIk and IncN plasmids. NDM-1 was found in co22 and co68. Moreover, the NDM-1 in co22 was related only to the IncA/C plasmids. Meanwhile, the NDM-1 in co68 was not related to any Inc plasmids (Table 4 and Figure 9C). NDM-1 was closely related to co18/co22/co68 communities including plasmids carrying CTX-M and KPC. Thus, co22/co68 have the most potential for the dissemination of AMR genes in *Enterobacteriacea.*

**Table 4:** Distribution of Inc types, antimicrobial resistance genes, and communities.

Inc type	CTX-M			KPC				NDM-1	
	M-1	M-2	M-9	2	3	4	10	co22	co68
FII	7		8						1
FIIk	4			5	2				1
N	2	1	3	1	1	1			
A/C			1					6	
L/M	2					1			2
I1	3								
FIB-M	1								1
FIA	1								
FIIS			1						
HI1									1
HI2	1								
K			1						
R				1					
Not Assigned	5	4	4	4	1		1		12
Total	26	5	18	11	4	2	1	6	18

## 3. Experimental Section

### 3.1. Download and Selection of Complete Plasmids Sequences

Complete plasmid candidates were downloaded in GenBank format from NCBI using the following key words: ((bacteria[Organism]) AND complete[Title]) AND plasmid[Title].

Among the downloaded sequences, complete, non-provisional sequences with more than 2000 bp sequences were used for the following analysis. For the remaining sequences, the protein sequences were extracted according to the “FEATURES” information.

### 3.2. Network Analysis

We performed the blastp searches [28] to identify orthologous genes between plasmids with parameter the -F F, -g T. The hits with e-values less than 1E-10, cover ratios more than 90% and length difference of 0.9 < query/subject < 1.1 with several identity cut-off values (100, 99, 90, 80, 70, 60, and 50%) were regarded as shared genes. We also searched for shared proteins using the uclust program v6.0.307 [29] with the following parameters after sorting by sequence length: -cluster_smallmem -minsl 0.9 -minqt 0.9 -maxqt 1.1 -query_cov 0.9 -target_cov 0.9. Subsequently, network analyses were performed using the R igraph library [21]. The plasmid communities were detected using the edge.betweenness.community, multilevel.community, label.propagation.community, infomap.community, walktrap.community, and fastgreedy.community methods in the igraph library by default parameter settings. For instance, if two plasmids share *n* genes, the edge weight parameter will be defined as *n*. Based on the intensity of the detected weight parameter on each edge, CCs were summarized into each community by the above method.

### 3.3. Inc Typing and Searching for Mobile Elements

Inc types were inferred from blastn searching with e-values less than 1E-10, cover ratios more than 90% against the PCR amplicons based on primer sequences for PBRT replicon typing [26].

### 3.4. Detection of CTX-M Genes

The CTX-M genes were prepared as follows. Candidate sequences for the CTX-M genes were retrieved from fasta formatted NCBI data using the key word “CTX-M”. The sequences with lengths of at least 291 aa that did not belong to synthetic constructs (*i.e*., lacking the “[synthetic construct]” search term) were extracted from the candidate sequences and were entered into the CTX-M database. We then performed a blastp search of the plasmid genes against the CTX-M database. The genes with greater than 90% identity for more than 280 aa in length were considered plasmid CTX-M genes. Finally, the plasmid CTX-M genes and CTX-M database sequences were aligned using the mafft program [30]. A phylogenetic tree was then constructed using the MEGA 5 program [31]. The groupings of the plasmid CTX-M genes were inferred according to the phylogenetic tree and the review from Bonet *et al.* [27].

### 3.5. Detection of Other Antibiotic Resistance Genes

We performed blastp searches for all of the plasmid genes against the ARDB + CARD database [32,33]. The plasmid proteins having the top hit to one of the following accessions with e-values less than 1E-10 were regarded as KPC genes: AAG13410, AAS01762, AAU06362, ABX11263, AF297554, AF395881, AY034847, EU555534, EU729727, FJ234412, GQ140348, HM066995, HQ641421, YP_002286834, YP_002286834, YP_002286834, YP_002286834, YP_002286834, YP_002286834, YP_002286834, YP_002286834, YP_002286834, YP_002286981, and YP_002286981. Accordingly, those plasmid proteins with a top hit being the following accessions with e-values less than 1E-10 were considered NDM-1 genes: HQ328085 and HQ451074. Plasmids with no significant hits within this database were regarded as having no AMR genes.

## 4. Conclusions

We conducted a plasmidome network analysis for all of the available complete bacterial plasmids. Most of the plasmids were connected to a single LCC, which mainly consisted of plasmids from *Bacilli* and *Gammaproteobacteria*. The LCC was subdivided into dozens of communities, which consisted of plasmids from a mostly single class of bacteria. Such community network analysis facilitate displaying possible recent HGTs like a class 1 integron, *str* and *tet* resistance markers between communities. As well as the class 1 integron, the CTX-M ß-lactamase can be identified in multiple communities. Therefore further dissemination could be generated either by plasmid transfer itself or transposition of the CTX-M ß-lactamase into other broad-host range plasmids “en bloc”, suggesting that this community network analysis could propose crucial information for the manner of dissemination in AMR. Intriguingly, HGT was noted between different phyla (*i.e.*, *Staphylococcus* and *Pasteurellaceae* (*P. multocida* and *H. parasuis*)), suggesting that both organisms can acquire AMR genes from closely contacting each other. The distributions of the Inc-type and AMR genes implied that such genes generally circulate within communities composed of typical bacterial taxa. This plasmidome network analysis under very strict parameter settings provides remarkable discrimination power for plasmid-related recent HGT. It also provides a large-scale analysis of plasmid associations, improving our understanding of current plasmid dissemination and evolution among bacterial communities.
